# Psammaplysins: Insights from Natural Sources, Structural Variations, and Pharmacological Properties

**DOI:** 10.3390/md20110663

**Published:** 2022-10-25

**Authors:** Diaa T. A. Youssef, Lamiaa A. Shaala

**Affiliations:** 1 Department of Natural Products, Faculty of Pharmacy, King Abdulaziz University, Jeddah 21589, Saudi Arabia; 2 Natural Products Unit, King Fahd Medical Research Center, King Abdulaziz University, Jeddah 21589, Saudi Arabia; 3 Suez Canal University Hospital, Suez Canal University, Ismailia 41522, Egypt

**Keywords:** marine alkaloids, 1,6-dioxa-2-azaspiro[4.6]undecane backbone, natural occurrence, isolation, structural variations, semisynthesis, pharmacological properties

## Abstract

Marine natural products (MNPs) continue to be in the spotlight in the global drug discovery endeavor. Currently, more than 32,000 structurally diverse secondary metabolites from marine sources have been isolated, making MNPs a vital source for researchers to look for novel drug candidates. The marine-derived psammaplysins possess the rare and unique 1,6-dioxa-2-azaspiro [4.6] undecane backbone and are represented by 44 compounds in the literature, mostly from sponges of the order Verongiida. Compounds with 1,6-dioxa-2-azaspiro [4.6] undecane moiety exist in the literature under five names, including psammaplysins, ceratinamides, frondoplysins, ceratinadins, and psammaceratins. These compounds displayed significant biological properties including growth inhibitory, antimalarial, antifouling, protein tyrosine phosphatase inhibition, antiviral, immunosuppressive, and antioxidant effects. In this review, a comprehensive literature survey covering natural occurrence of the psammaplysins and related compounds, methods of isolation, structural differences, the biogenesis, and biological/pharmacological properties, will be presented.

## 1. Introduction

The oceans are considered the largest habitat on the Earth. It is reported that the total number of the identified marine species is 240,000 [1], and it is estimated that there are 1.4–1.6 million marine species on Earth [2]. Another, more recent estimate for marine species is about a half lower (0.7–1.0 million) [3]. The reason for the enormous range in the estimated number of marine species lies in the deficiency of the data about the diversity of marine microbes and other microscopic organisms. For example, sufficient data are available on marine mammals and fishes, while enough and satisfied data about the huge microbial diversity and the phytoplankton in the oceans still need to be revealed. Alone in Europe, it is estimated that about 41,000–56,000 species exist with 5000–20,000 species yet to be identified. Annually, there are about 1000–1500 new marine species documented.

Marine invertebrates are the most diverse group of marine life that exist in the oceans with highest biological and chemical diversity. A total of 38,925 marine-derived natural products, published in 38,645 articles, were identified from marine organisms [4]. Such chemical biodiversity is attributed to the fact that these compounds are produced by mostly sessile organisms present in the marine environment. These sessile organisms are extremely susceptible to be attacked by predators. Marine invertebrates, such as sponges, tunicates, bryozoans, gorgonians, and soft corals evolve chemicals as a defense mechanism against highly mobile predators. Marine sponges are one of the most productive phyla of marine invertebrates and considered as an outstanding source of biologically active secondary metabolites [5].

Members of the Phylum Porifera and their associated microbes represent the largest reservoir and supplier of secondary metabolites. Primitive and sessile animals, such as sponges, developed survival strategies depending on the production of defensive secondary metabolites for their own protection against different predators, fouling organisms, and invasion by different microbes and pathogens. Sponges are classified in four major classes including Calcarea, Demospongiae, Hexactinellida, and Homoscleromorpha. The chemical structural diversity of the secondary metabolites of the sponges includes several classes, such as alkaloids, macrolides, peptides, steroids, terpenoids, polyketides, and many others. [6].

It was proven that the sponge-derived secondary metabolites produce an enormous array of antitumor, antiviral, anti-inflammatory, antibiotic, and other bioactive molecules that have the potential for therapeutic use. Studies have shown that different compounds affect the targeted disease through different modes of action. Chemical entities that can act as transcription factor inhibitors may be effective against both viral infections and malignant neoplasms. Most bioactive metabolites from sponges have proven to inhibit specific enzymes, which often mediate or produce mediators of intercellular or intracellular messengers involved in the development of diseases [7].

Amongst different orders of marine sponges, the order Verongiida is considered as a reservoir of brominated tyrosine-derived secondary metabolites. This order belongs to the kingdom Animalia, phylum Porifera, class Demospongia, and subclass Verongimorpha. According to the World Porifera Database, order Verongiida includes five families viz. *Aplysinellidae, Ernstillidae, Aplysinidae, Ianthellidae,* and *Pseudoceratinidae.*

Members of the order Verongiida attracted researchers of the marine natural products community over the past 65 years due to the large number of bioactive bromotyrosine-derived alkaloids that they produce [5]. Bromotyrosine-derived alkaloids display significant chemical diversity and offer effective chemical defense for these organisms against predators in the ocean [8,9] and the fouling organisms [10,11].

Bromotyrosine derivatives of the order Verongiida include several classes, such as spirooxepinisoxazolines, mono- and bis-configurated spiroisoxazolines, dibromocyclohexadienes, brominated phenolics, verongiaquinols, verongiabenzenoids, oxime disulfides, brominated oximes, bromotyramines, bromotyramine oximes, bastadins, and hemibastadins. Additional chemical classes that are not of bromotyrosine biosynthetic origin in this order are indole alkaloids, pyrroles, quinolines, hydroquinones guanidines, benzofurans isoprenoids, benzonaphthyridines, sesquiterpenoids, sesterterpenoids, merosesquiterpenoids, and macrolides [12].

The bromotyrosine-derived compounds are considered as a class of interest due to their structural diversity and pharmacological and biological importance [13,14,15,16]. Prominent members of the bromotyrosine derivatives include psammaplins, disulfide-linked compounds, that were first isolated from an unidentified specimen of *Psammaplysilla* Keller, 1889 (=*Pseudoceratina* Carter, 1885) [17]. These compounds have stimulated further and deeper investigations on other Verongiid sponges as well as the synthesis of targeted anti-cancer drug analogs [18,19,20]. The bromotyrosin-derived compounds of the Verongiid sponges display huge pharmaceutical and biomedical potential, with many viewed as being promising targets within the preclinical pipeline. Preclinical assays on bromotyrosines have highlighted many candidates for antiplasmodial [21,22], antimicrobial [22,23,24,25,26,27,28,29,30,31,32], antioxidant [27,28], anti-invasion, and antimigratory [33,34,35], parasympatholytic [36], as well as compounds that affect the central nervous system [23,29,37,38]. These significant and broad-spectrum activities have provided much motivation for further investigations of the members of this order for the exploration of its secondary metabolites and biomedical importance.

To date, more than 633 natural products, mostly bromotyrosine-derived, are reported from over 43 different species of the order Verongiida in the literature [12]. Among these, forty-one bromotyrosine alkaloids possessing the 1,6-dioxa-2-azaspiro [4.6] undecane skeleton have been reported from marine sponges of the Verongiida, including members of genera *Aplysinella, Psammaplysilla, Pseudoceratina*, and *Subarea*, and three compounds from the order Dictyoceratida (including the genera *Dysidea* and *Hyattella*).

Compounds with 1,6-dioxa-2-azaspiro[4.6]undecane moiety (Table S1) exist in the literature under five names *viz.* psammaplysins, ceratinamides, frondoplysins, ceratinadins and psammaceratins. This review will cover the natural occurrence, isolation protocols, structural differences, the biosynthesis, and biological properties of the reported compounds with 1,6-dioxa-2-azaspiro[4.6]undecane moiety between 1983 and 2022. For the convenience for the reader, the name “psammplyisn” will be used as a general name for this class in this review.

## 2. The Beginning

**Figure 1:**
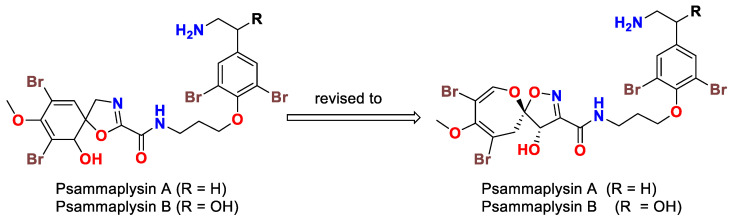
Initial proposed (**left**) and revised structures (**right**) of psammaplysins A and B.

The isolation of psammaplysin series started in 1983 by the isolation of two spirooxepinisoxazoline type dibromotyrosine derivatives, psammaplysins A and B, from the Red Sea sponge *Psammaplysilla purpurea* [39]. Initially, the planar structures of psammaplysins A and B were determined as having a spiro [4.5] oxazadecane backbone after interpretation of ^1^H and ^13^C NMR spectral data of the compounds and alkaline degradation of psammaplysin A (Figure 1) [39]. In 1985, Scheuer’s group revised the basic skeleton of psammaplysins A and B from spiro[4.5]oxazadecane backbone to a spiro[4.6]dioxazundecane moiety (Figure 1) after analysis of the 2D ^13^C–^13^C connectivity of psammaplysin A and single-crystal X-ray diffraction studies on psammaplysin A acetamide acetate [40].

## 3. The Biosynthesis of Psammaplyisns

**Figure 2:**
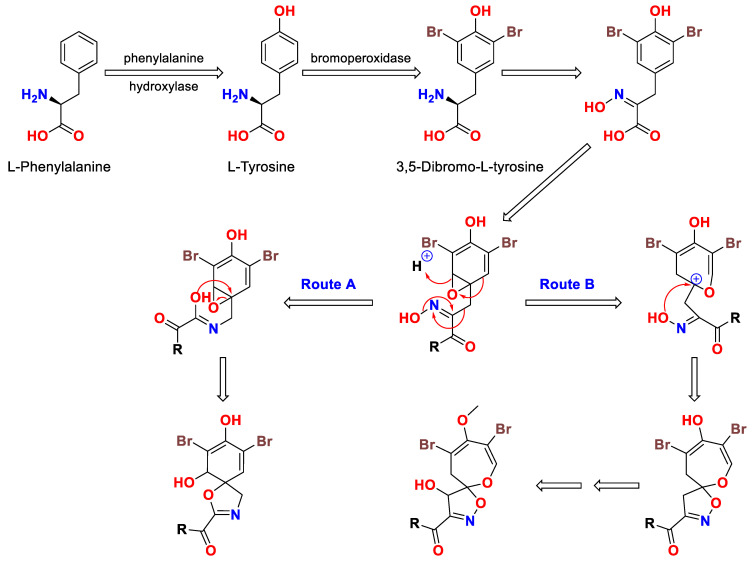
Proposed biosynthesis of 1-oxa-3-azaspiro[4.5]decane and 1,6-dioxa-2-azaspiro[4.6]undecane backbones (modified from [40]).

In 1985, the biosynthetic pathway of the 1,6-dioxa-2-azaspiro [4.6] undecane skeleton from 3,5-dibromo-l-tyrosine was proposed by Roll et al. [40]. The biosynthesis of the 1,6-dioxa-2-azaspiro[4.6]undecane moiety of psammaplysins may proceed through an oximino epoxide as shown in Figure 2. A Beckmann type rearrangement concomitant with an epoxide ring opening (Route A) will lead to the basic moiety (1-oxa-3-azaspiro [4.5] decane) of aerothionin’s family. If an epoxide ring opening occurs, this will leads to ring enlargement, resulting in the basic subunit (1,6-dioxa-2-azaspiro[4.6]undecane) of psammaplysin family (Route B) (Figure 2) [40].

## 4. The Chemistry of Psammaplysins

**Figure 3:**
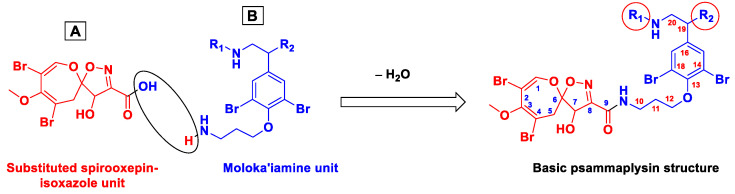
Structural subunits of psammaplysins.

The psammaplysin backbone is composed of two dibrominated moieties, 8,10-dibromo-4-hydroxy-9-methoxy-1,6-dioxa-2-azaspiro[4.6]undeca-2,7,9-triene-3-carboxylic acid (subunit A), and 3-(4-(2-aminoethyl)-2,6-dibromophenoxy)propan-1-amine subunit (subunit B, moloka’iamine), linked together through an amidic linkage between the carboxylic moiety (C-9) of the substituted spirooxepinisoxazoline unit and the terminal amino group at C-10 of the moloka’iamine (Figure 3). Interestingly, moloka’iamine (subunit B) and its substituted derivatives were reported from several Verongiid sponges, but there is no single report in the literature about the existence or isolation of the separated dibrominated spirooxepinisoxazoline moiety (subunit A). The substituted and dibrominated spirooxepinisoxazoline unit has been always associated with the moloka’iamine moiety via an amidic moiety. This can be explained by the necessity of such combination as a defense tool for sponges’ survival against predators [41].

## 5. The Absolute Configuration of Psammaplysins

**Figure 4:**
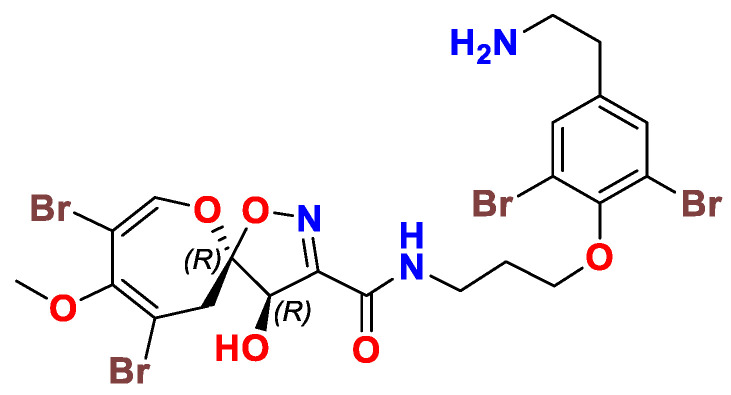
Structure of psammaplysin A showing the 6*R*,7*R* configuration.

Later in 2015, the absolute configuration of the stereogenic carbons (C-6 and C-7) of psammaplysin A was verified as 6*R* and 7*R* (Figure 4), respectively, through using experimental and calculated electronic circular dichroism (ECD) data and NMR analysis of MPA esters prepared from the acetamide derivative of psammaplysin A. Detailed conformational analyses of a truncated model compound of psammaplysin A with an *in vacuo* method and with the Polarizable Continuum Model (PCM) solvent model for MeOH have identified the major conformers and factors governing the ECD spectrum of psammaplysin A. The correlation of the ECD data of psammaplysin A will allow future configurational assignments of related psammaplysin analogs on the basis of comparison their ECD spectra [42].

Since all reported natural psammaplysins in this review possess a negative sign of optical rotation, we therefore assume that all reported psammaplasyins possess the same biosynthetic pathway and therefore have absolute configuration at C-6 and C-7 as 6*R* and 7*R*. Accordingly, all structures in this review have been drawn with the 6*R*,7*R* configuration regardless of their original drawings in the original manuscripts.

## 6. Purification of Psammaplysins

**Figure 5:**
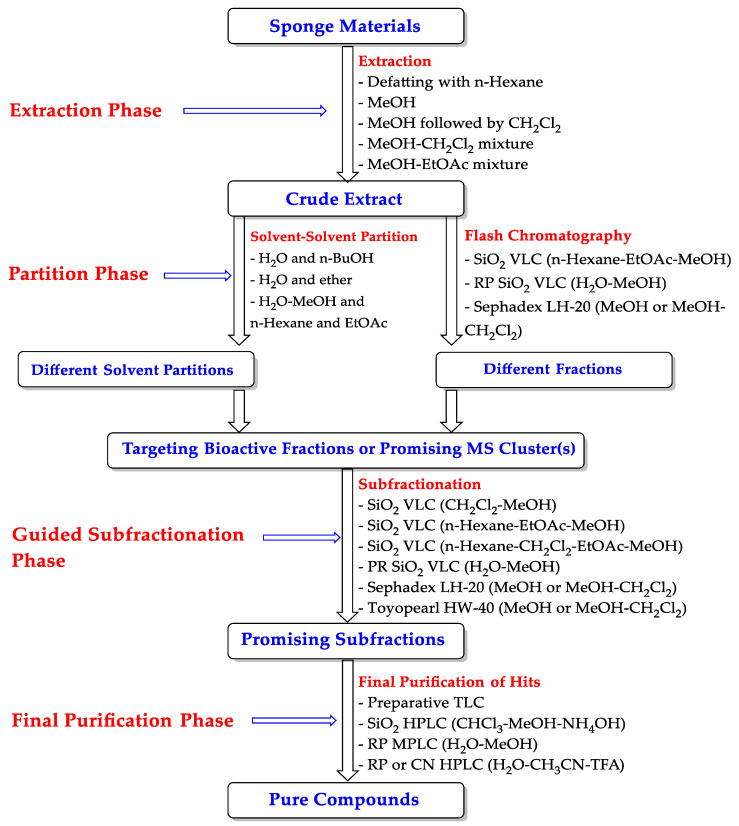
General purification scheme of psammaplysins.

As discussed above, 44 psammaplysins are obtained from different sponges using multiple chromatographic techniques [39,41,43,44,45,46,47,48,49,50,51,52,53,54]. To make it easier for the readers, a general purification protocol was outlined that contains the most important steps for the purification of these compounds (Figure 5).

Figure 5 shows that the purification process contains four main phases including the extraction phase (I), the partition phase (II), subfractionation of targeted fraction(s) phase (III), and finally the purification of hits (IV).

At the beginning, phase I includes prior extraction of the sponge materials with n-hexane for the removal of undesired lipophilic materials followed by extraction with desired solvent(s). Otherwise, the materials are directly extracted with one solvent (MeOH), or a mixture of solvents (MeOH-CH_2_Cl_2_, MeOH-CHCl_3_) or sequential extraction with different solvents (MeOH followed by CH_2_Cl_2_, etc.) (Figure 5). The partition phase (II) starts with either solvent-solvent partitioning of the crude extract using different immiscible solvents or mixture of solvents or applying direct flash VLC chromatography on either resin, normal- or reversed-phase silica using a variety of organic and aqueous solvents (Figure 5). In some cases, phase III starts with targeting specific bioactivity using suitable screen or targeting a specific class of the compounds using LC-MS clusters to target the multi-brominated compounds. Otherwise, direct sub-fractionation of the extracts’ fractions on resin, normal- or reversed-phase silica was performed to prepare the targeted subfractions for the final purification (Figure 5). The final purification of the targeted compounds was usually achieved by MPLC or HPLC on normal-, reversed-phase silica, or CN columns using a variety of eluting solvents (Figure 5).

## 7. Natural Occurrence

As mentioned above, in 1983 Kashman’s group reported the isolation of psammaplysins A (**1**) and B (**2**) (Table 1) from the Red Sea sponge *Psammaplysilla purpurea* [39]. Almost a decade later, psammaplysin C (**3**) (Table 1), an *N*-methylated derivative of psammaplysin B, was identified in 1992 from the sponge *Druinella* (=*Psammaplsilla*) *purpurea*, family Druinellae (Aplysinellidae), order Verongida which was collected from the shallow reef waters off Makaluva Island of the Fiji Island Group in the South Pacific [43]. In 1993, the new analogs psammaplysins D (**4**) and E (**5**) (Table 1), along with psammaplysin A (**1**), were purified from a new species of the sponge *Aplysinella* (Family Aplysinellidae) collected from a vertical coral wall at Pingelap Atoll, Micronesia [44].

In 1996, the identification of additional psammaplysin derivatives continued under the name of “ceratinamides”. Ceratinamides A (**7**) and B (**8**) (Table 1) were obtained from the sponge *Pseudoceratina purpurea* collected from Hachijo Island [45]. In 1997, the new psammaplysin derivative, psammaplysin F (**10**) (Table 1), along with psammaplysins A-C (**1**–**3**) and E (**5**), was isolated from an undescribed species of *Aplysinella* sponge [46]. The new psammaplysin G (**11**) (Table 1), along with psammaplysin F (**10**), was purified from a non-Verongiid sponge, *Hyattella* sp. (Spongiidae) in 2010 [47].

In 2011, the identification of a new analog, psammaplysin H (**12**) (Table 1), along with psammaplysins F (**10**) and G (**11**) was reported from the marine sponge *Pseudoceratina* sp. [48]. Psammaplysins I (**13**) and J (**14**) (Table 1), new psammaplyisn analogs, were reported in 2012 from the organic extract of the sponge *Suberea* sp. (family: Aplysinellidae) collected at Black Coral Kingdom, Guam along with psammaplysins A (**1**) and B (**2**) [49].

The identification of psammplysin derivatives was continued by Mudianta group in 2012 through investigation of the Balinese marine sponge *Aplysinella strongyalata* collected by in Tulamben Bay, Bali. In addition to the reported psammaplysins A (**1**), B (**2**), D (**4**), E (**5**) and ceratinamides A (**7**) and B (**8**), 21 new psammplysin analogs, namely 19-hydroxypsammaplysin E (**6**) (Table 1), psammaplysin K (**15**), psammaplysin K dimethoxy acetal (**16**), psammaplysins L (**17**), M (**18**), N (**19**), O (**20**), P (**21**), 19-hydroxypsammaplysin P (**22**), psammaplysin Q (**23**), 19-hydroxypsammaplysin Q (**24**), psammaplysin R (**25**), psammaplysin S (**26**), 19-hydroxypsammaplysin S (**27**), psammaplysin T (**28**), 19-hydroxypsammaplysin T (**29**), psammaplysin U (**30**), 19-hydroxypsammaplysin U (**31**), psammaplysins V (**32**) and W (**33**) and 19-hydroxypsammaplysin W (**34**) (Table 1), were isolated and characterized [50].

In 2013, the identification of four new psammaplysin analogs, psammaplysin X (**35**), 19-hydroxypsammaplysin X (**36**), psammaplysin Y (**37**) and 19-hydroxyceratinamide A (**9**) (Table 1), along with psammaplysins A (**1**), B (**2**), D (**4**), E (**5**), and 19-hydroxypsammaplysin E (**6**) from the marine sponge *Suberea* sp. collected offshore of Chuuk, Federated States of Micronesia was reported [51].

In 2019, bioassay-directed fractionation of the extract of the Red Sea sponge *Aplysinella* sp. resulted in purification of two additional psammaplysin analogs, psammaplysin Z (**38**) and 19-hydroxypsammaplysin Z (**39**) (Table 1) along with psammaplysins A (**1**) and E (**5**) [41]. In 2018, the name “ceratinadins” was given to two new psammaplysin analogs, ceratinadins E (**40**) and F (**41**) (Table 1), that were isolated from the marine sponge *Pseudoceratina* sp. collected in Okinawa, Japan, along with psammaplysin F (**10**) [52].

In 2019, two psammaplysin analogs, frondoplysins A (**42**) and B (**43**) (Table 1), were reported from a second non-Verongiid marine sponge, *Dysidea frondosa,* which was collected from the South China Sea [53].

Finally, in 2021, the name “psammaceratin” was given to the first psammaplysin dimer in the series. Psammaceratin A (**44**) (Table 1) was reported from the Red Sea sponge *Pseudoceratina arabica* collected in the Red Sea [54]. 

## 8. Structural Variations

Comparing to psammaplysin A, psammaplysin C (**3**) represents the first *N*-methylated derivative of the psammaplysin series. In addition to an OH moiety at C-19, psammaplysin D (**4**) possesses an isopentadecanoyl residue at the terminal amine of the compound through an amidic linkage. Furthermore, psammaplysin E (**5**) possesses an unprecedented cyclopentene-dione backbone linked to the terminal amine of the compound [44].

Interestingly, ceratinamide A (**7**) is the first compound with an *N*-formyl functionality at the terminal amine, while ceratinamide B (**8**) contains a 13-methyltetradecanoic acid moiety through an amidic linkage with the terminal amino group of the compound [45].

Psammaplysin F (**10**) represents the second *N*-methylated psammaplysin analog in this series [46]. In addition to a terminal *N*-methyl moiety, psammaplysin G (**11**) possesses an urea moiety at the terminal amino group. Further, psammaplysin G is the first psammaplysin analog with a tertiary amine in this series.

Psammaplysin H (**12**) possesses an *N*-trimethyl substitution, being the first quaternary psammaplysin analog in this series.

Surprisingly, psammaplysins I (**13**) and J (**14**) are lacking the bromine atom at C-18 of the moloka’iamine part of the molecule, being the first psammaplysin analogs with only three bromine atoms.

Psammaplysins K (**15**), K dimethoxy acetal (**16**), L (**17**), M (**18**), and 19-hydroxypsammaplysin E (**6**) display discrepancy in the structural unit attached to C-16 of the aromatic moiety or the terminal amine of the compound. For example, psammaplysin K possesses an aldehydic moiety at C-16 instead of the ethylamine part, while a dimethoxy acetal moiety exists at C-16 in psammaplysin K dimethoxy acetal. Further, psammaplysin L contains a 2-oxazolidinone moiety at C-16, while a glycolamide moiety appears at the terminal amine in psammalysin M. Finally, 19-hydroxypsammaplysin E contains the previously reported cyclopentene-dione moiety in psammaplysin E in addition to an OH at C-19 [50].

Psammaplysins U (**30**), 19-hydroxypsammaplysin U (**31**), V (**32**), W (**33**) and 19-hydroxypsammplysin W (**34**) possess a monoenoic fatty acid moiety. In psammaplysin U and 19-hydroxypsammaplysin U, an isobranched fatty acid moiety exists, while the other psammaplysins (V, W, and 19-hydroxypsammplysin W) have straight chain fatty acid moiety [50].

Psammplysin X (**35**) and 19-hydroxypsammaplsyin X (**36**) have the unique 4-chloro-2-methylenecyclopentane-1,3-dione moiety at the terminal amino group, representing the first chlorinated psammaplysin analogs in this group. On the other hand, in psammaplysin Y (**37**) the existence of the rare 2-methylenecyclopentane-1,3-dione moiety at the terminal amine was reported. Further, 19-hydroxyeratinamide A contains, beside a terminal *N*-formyl moiety, an OH at C-19 [51]. Additionally, psammaplysin Z (**38**) and 19-hydroxypsammaplysin Z (**39**), and possesses an urea moiety on the terminal amino groups [41].

On the contrary from psammaplysin A, ceratinadins E (**40**) and F (**41**) possess two and three moloka’iamine units, respectively. Thus, ceratinadins E and F possess a total of six and eight bromine atoms, respectively. Another feature in both compounds is the connectivity of all moloka’iamine moieties through *N*-methylated urea and the *N*-methylation of all terminal amines of the moloka’iamines’ moieties [52]. In addition to the C-19 OH moiety, frondoplysins A (**42**) and B (**43**) possess an unprecedented bioconjugates of a meroterpene moiety attached to the terminal amine of the psammaplysin backbone making these compounds the first example in this group with a terpene backbone via “N-C” linkage. Finally, psammaceratin A (**44**) is composed of two units of psammaplysin A connected together via an unprecedented (2*Z*,3*Z*)-2,3-bis(aminomethylene)succinamide moiety, thus representing the first dimer among this class [54].

## 9. Semisynthetic Analogs of Psammaplysins

**Figure 6:**
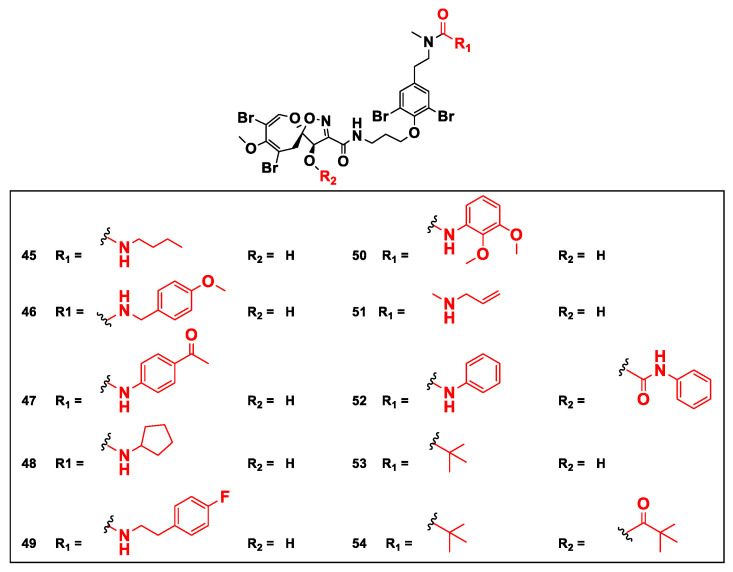
Structures of urea (**45**–**52**) and amide (**53** and **54**) semisynthetic derivatives of **10** displaying added moieties [55].

In 2020, a series of psammaplysin F semisynthetic derivatives including urea (**45**–**52**) and amide (**53** and **54**) analogs (Figure 6) was prepared. 

This library of compounds was investigated for their effect on cell cycle distribution and changes to cancer metabolism in LNCaP prostate cancer using a multiparametric quantitative single-cell imagining approach [55].

## 10. Pharmacological Properties

### 10.1. Compounds with Antimicrobial Properties

Psammaplysins A (**1**) and B (**2**) have been reported to show in vitro activity towards gram positive bacteria as well as *E. coli* [39]. In addition, psammaplysin A (**1**) has found to possess antibacterial activity against *Flavobacterium marinotypicum* with an inhibition zone of 10 mm at a concentration of 10 µg/disc [45]. Furthermore, Psammaplysins A (**1**) and B (**2**) have been described to inhibit the *Mycobacterium tuberculosis* detoxification enzyme mycothiol-*S*-conjugate amidase in a fluorescence-detected assay [56]. Furthermore, psammaplysins F (**10**) and H (**12**) have been found to inhibit the growth of six Gram-positive strains, *S. aureus* NCTC 6571, *S. aureus* 1H, *E. facecalis* NCTC-775, B. cereus NCTC-7464, MRSA MW2 and MRSA USA-300 [56] (Table 2).

### 10.2. Compounds with Growth Inhibition and Cytotoxic Activities

Psammaplysin A (**1**) has been reported as a growth inhibitor of many cancer cell lines including, HCT-116, HCT-15 (colon cancer), PC-3 (prostate cancer), ACHN (renal cancer), MDA-MB-231 (breast cancer), NUGC-3 (stomach cancer), NCI-H23 (lung cancer), and Hela (Cervical) [41,43,51,54] (Table 3).

Similarly, psammaplysin B (**2**) was found to inhibit the growth of several cell lines, including HCT-116, HCT-15 (colon cancer), PC-3 (prostate cancer), ACHN (renal cancer), MDA-MB-231 (breast cancer), NUGC-3 (stomach cancer), and NCI-H23 (lung cancer) [43,51] (Table 3).

Psammaplysin C (**3**) has been reported to inhibit the growth of HCT-116 cell line [43] (Table 2). Likewise, psammaplysin D (**4**) has been reported to inhibit the growth of HCT-15 (colon cancer), PC-3 (prostate cancer), ACHN (renal cancer), MDA-MB-231 (breast cancer), NUGC-3 (stomach cancer), and NCI-H23 (lung cancer) cell lines [51] (Table 3).

Psammaplysin E (**5**) has been reported as a potent growth inhibitor of several cancer cell lines, including KB (human oral, epidermoid carcinoma), LoVo (human colon, adenocarcinoma), HCT-15, PC-3, ACHN, MDA-MB-231, NUGC-3, and NCI-H23 [41,44,45,51]. In addition, it has been found that psammaplysin E possesses a potent antimigratory effect against MDA-MB-231 and Hela cells [41] and has moderate immunosuppressive activity as well [44] (Table 3).

Psammaplysin F (**10**) has been reported as a moderate inhibitor of HEK293 mammalian cell line [47] (Table 3).

Psammaplysin X (**35**) and 19-hydroxypsammaplysin X (**36**) were reported to inhibit the growth of HCT-15, PC-3, ACHN, MDA-MB-231, NUGC-3, and NCI-H23 cancer cell lines [51] (Table 3).

Psammaplysin Z (**38**) and 19-hydroxypsammaplysin Z (**39**) were found to inhibit the growth of MDA-MB-231 and HeLa cancerous cell lines [41] (Table 3).

Finally, psammaceratin A (**44**) has been reported to inhibit the growth of MDA-MB-231, Hela, and HCT116 cell lines [54] (Table 3).

From the above results, it could be concluded that the growth inhibition of the psammaplysins towards cancerous cell lines suggests that the spirooxepinisoxazoline ring system is an essential element for the activity. The *N*-terminal substitution with a cyclopentene-dione moiety, as in psammaplsyins E (**5**) and 19-hydroxypsammaplysin E (**6**), or 4-chloro-2-methylenecyclopentane-1,3-dione moiety, as in psammaplysin X (**35**) and 19-hydroxypsammaplysin X (**36**), increases the activity. Further, introduction of 19-OH group, as in psammaplysin B (**2**) versus psammaplysin A (**1**), diminishes the activity (Table 3).

On the contrary, psammaplysin D (**4**), however, lacked activity (GI_50_ > 10 μM), which might be explained by its high lipophilicity. Furthermore, the existence of a terminal *N*-methyl group, as in psammaplysin F (**10**), or an urea moiety, as in psammaplysin Z (**38**), diminished the growth inhibition effect (Table 3).

### 10.3. Compounds with Antimalarial Activities

19-Hydroxypsammaplysin E (**6**), psammaplysin K (**15**), psammaplysin L (**17**), psammaplysin M (**18**), psammaplysin N (**19**), 19-hydroxypsammaplysin P (**22**), psammaplysin T (**28**), and psammaplysin V (**32**) have been evaluated for their antimalarial activity, at 10 μM, against the chloroquine-sensitive *Plasmodium* *falciparum* 3D7 malaria parasite line. Only 19-hyroxypsammaplysin E (**6**) was found to have antimalarial effect against this strain [50] (Table 4).

Similarly, psammaplysin F (**10**) has been reported to inhibit chloroquine-sensitive (3D7), Dd2 [47], the drug-resistant (K1) and drug-sensitive (FCR3) strains of *P. falciparum* [52] (Table 4). Furthermore, psammaplysin G (**11**) has been found to inhibit the chloroquine-resistant (Dd2) *P. falciparum* strain without any toxicity towards the HEK293 cell line [47]. Likewise, psammaplysin H (**12**) was described to have a potent antiplasmodial activity against 3D7 strain with an excellent selectivity index [48] (Table 4).

Finally, ceratinadins E (**40**) and F (**41**) were reported to show antiplasmodial activities against the drug-resistant (K1) and drug-sensitive (FCR3) strains of *P. falciparum*. Moreover, ceratinadin E (**40**) was found to display higher selectivity indices (SI) than ceratinadin F (**41**) [52] (Table 4).

The antimalarial evaluation of 13 psammaplysins analogs clearly shows that psammaplysin F (**10**) is the most potent active compound against Dd2 strain, while ceratinadin E (**40**) possesses a greater antimalarial activity towards K1 strain and a better selectivity index than psammaplysins F (**10**). The addition of a terminal *N*-methyl, as in psammaplysin F (**10**), enhances the activity. However, ceratinadin F (**41**), which possesses several *N*-methyls, did not show significant antimalarial activity, which could be attributed to the high lipophilicity of the compound. Though the antimalarial activity against drug-resistant strains of *P. falciparum* is unknown, psammaplysin H (**12**), a quaternary analog with a trimethylamino group instead of a methylamino group, possesses a potent antimalarial activity and better selectivity against a drug-sensitive strain of *P. falciparum* than psammaplysins F (**10**) without any significant cytotoxicity against the HEK293 384 cell line [47,48,50] (Table 4).

Comparing the activities of psammaplysins F (**10**), G (**11**), and H (**12**) towards two mammalian cell lines (HEK293 and HepG2), psammaplysin H was found to display a minimal toxicity at the highest concentration tested (40 μM), giving this compound a parasite-specific selectivity index (SI) of >97. In contrast, psammaplysins G and F display higher toxicity to these cell lines with IC_50_ values between 3.71 and 18.96 μM, respectively. These preliminary structure–activity data suggest that full methyl-substitution of the terminal amine (*N*-quaternization) is essential for optimal antimalarial activity and better selectivity [48].

Likewise, the replacement of an urea, amine, or enamine derivative with a secondary amide group adversely affects the antimalarial activity. However, the higher lipophilicity (i.e., log P) and larger molecular weights associated with the amide analogs including psammaplysin M (**18**), psammaplysin N (**19**), 19-hyroxypsammaplysin P (**22**), psammaplysin T (**29**, and psammaplysin V (**32**) would also minimize the bioavailability [58], thus reducing the antimalarial effect.

### 10.4. Compounds with Antifouling Activities

When evaluated for their antifouling activity, psammaplsyins A (**1**), E (**5**), ceratinamides A (**7**) and B (**8**) have reported to inhibit the metamorphosis and settlement of the barnacle *B. Amphitrite* [45] (Table 5). The highest activities of psammaplysin A (**1**) and ceratinamide A (**7**) suggests the importance of a terminal amine or an *N*-formyl moiety for a maximum antifouling activity. Furthermore, ceratinamide A (**7**) was found to induce a larval metamorphosis of the ascidian *Halocynthia roretzi* [45] (Table 5).

### 10.5. Compounds with Other Reported Activities

Psammaplysin D (**4**) was reported to display anti-HIV towards the Haitian RF strain of HIV-I [44] (Table 5). Recently, psammaplysin F (**10**) was reported to increase the efficacy of the antitumor drugs bortezomib and sorafenib through regulation of the synthesis of stress granules [59] (Table 6).

Frondoplysins A (**42**) and B (**43**) were described to inhibit protein-tyrosine phosphatase IB (PTP1B). The compounds were found to have a higher activity than the positive control oleanolic acid [53] and thiazolidinediones [60] and were similar to benzofuran and benzothiophene biphenyls [61] (Table 6). Further, frondoplysin A was found to possess in vivo antioxidant activity in transgenic fluorescent zebrafish over five times stronger than that of vitamin C [53] without any cytotoxicity [53] (Table 6).

It has been described that psamaplysin F (**10**) andits urea semisynthetic analogs (**45**, **51**, **53** and **54**) strongly reduce the mitochondrial membrane potential (MMP). Further, it was found that psammaplysin F strongly affects the mitochondrial morphology and reduced the number of end points and branch points within the tubular structure of individual mitochondria, leading to visible fragmentation of the mitochondrial tubular network. These findings provide a strong rationale for more detailed mechanistic studies of psammaplysin F and derivatives as novel mitochondrial poisons [55].

## 11. Summary

**Figure 7:**
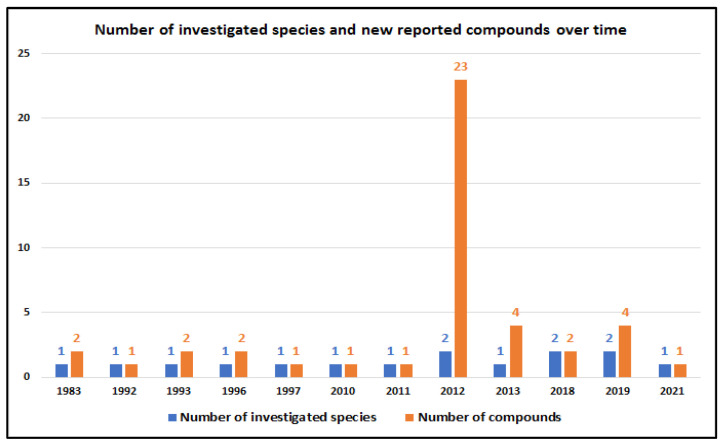
Number of investigated sponge species and number of reported new psammaplysins from species over time.

Since the first report of psammaplysins A and B in 1983, additional 42 compounds have been reported until now from 12 marine sponge species, including 10 Verongiid and two non-Verongiid sponges. The field was most active in the years 2012 (21 compounds), 2013 (four compound), and 2019 (four compounds) (Figure 7). Between 1992 and 2011 and in 2018 and 2021, either one or two new psammaplysin analogs were reported (Figure 7).

**Figure 8:**
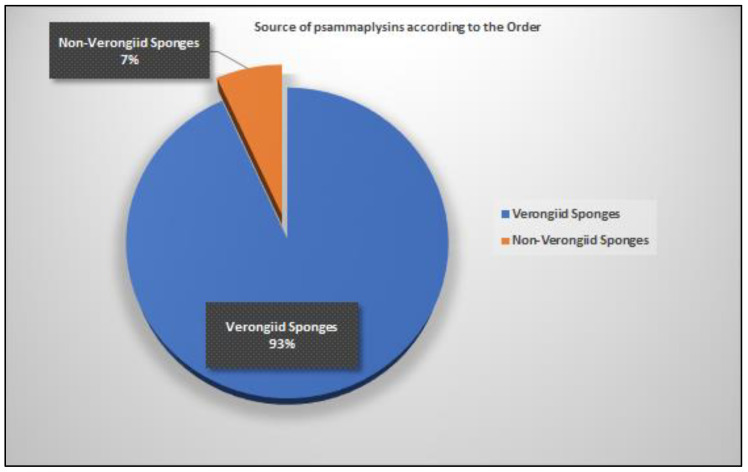
Number of reported psammaplysins derivatives per order.

**Figure 9:**
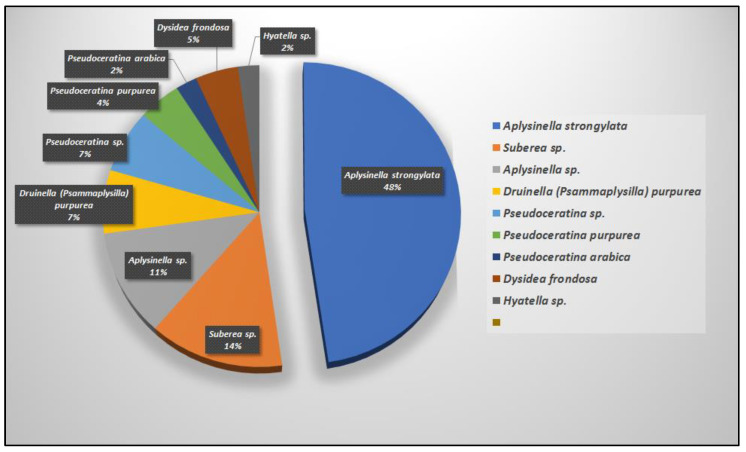
Number of reported psammaplysins (%) per sponge species.

The majority of the psammaplysins (41 compounds, 93%) come mainly from four Verongiid genera, including *Aplysinella* (26 compounds), *Pseudoceratina* (six compounds), *Suberea* (six compounds), and *Psammaplysilla* (three compounds) and non-Verongiid (three compounds) genera, including *Dysidea* (two compounds) and *Hyattella* (one compound) (Figure 8 and Figure 9).

**Figure 10:**
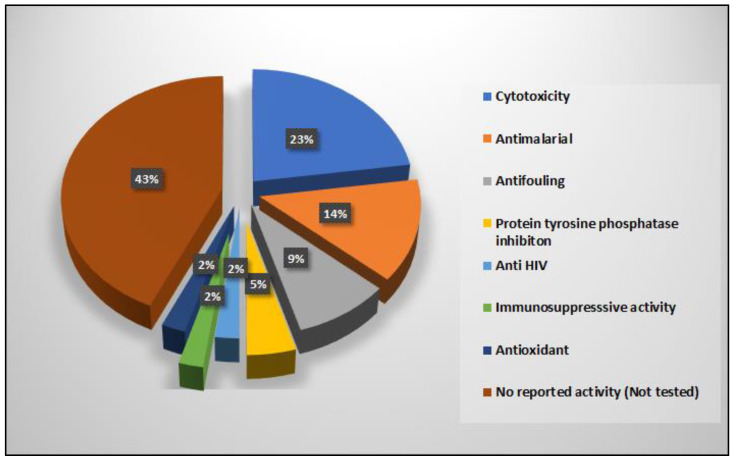
Number of psammaplysins (%) associated with biological activities.

As shown above, reported compounds with 1,6-dioxa-2-azaspiro[4.6]undecane moiety vary mainly in the presence of substituents at C-19 and/or the terminal amine, which greatly affected the biological properties of the compounds. From those, only 29 compounds have been found to possess variable bioactivities, such as cancer cell growth inhibition (12 compounds), antimalarial (6 compounds), antifouling (four compounds), antimicrobial (three compounds), and other activities (four compounds). The remaining 15 compounds are either inactive in one or more screens or are not evaluated at all (Figure 10).

In conclusion and from the data discussed before, some candidates with 1,6-dioxa-2-azaspiro[4.6]undecane skeleton exhibited significant antimalarial activity and growth inhibitory effects towards several human cancerous cell line making them attractive scaffolds for the development of potent antimalarial and antitumor leads.

## Figures and Tables

**Table 1:** Chemical structures and natural sources of psammaplyins.

Compound	Sponge Name	Reference
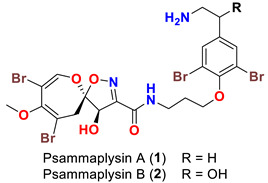	*Psammaplysilla purpurea*	[39]
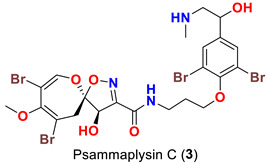	*Psammaplsilla purpurea*	[43]
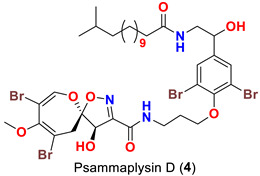	*Aplysinella* sp.	[44]
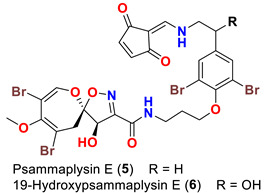	*Aplysinella* sp. *Aplysinella strongyalata*	[44,50]
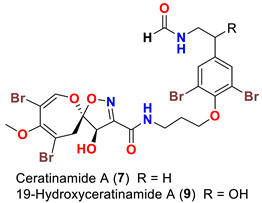	*Pseudoceratina purpurea* *Suberea* sp.	[45,51]
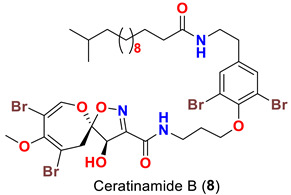	*Pseudoceratina purpurea*	[45]
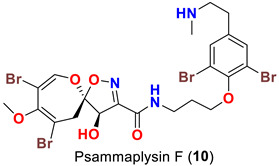	*Aplysinella* sp.	[46]
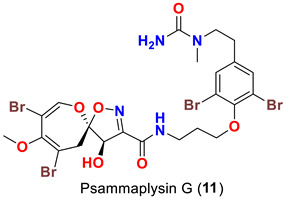	*Hyattella* sp.	[47]
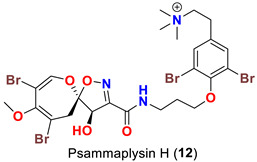	*Pseudoceratina* sp.	[48]
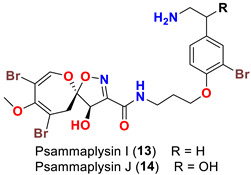	*Suberea* sp.	[49]
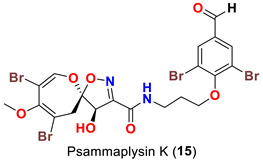	*Aplysinella strongyalata*	[50]
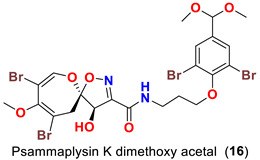	*Aplysinella strongyalata*	[50]
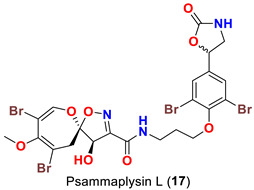	*Aplysinella strongyalata*	[50]
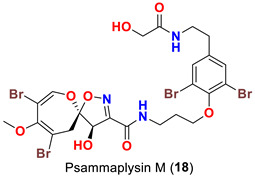	*Aplysinella strongyalata*	[50]
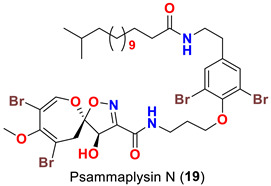	*Aplysinella strongyalata*	[50]
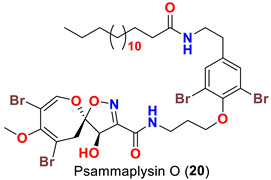	*Aplysinella strongyalata*	[50]
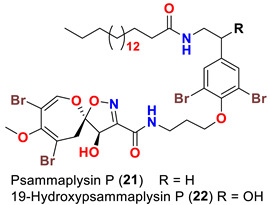	*Aplysinella strongyalata*	[50]
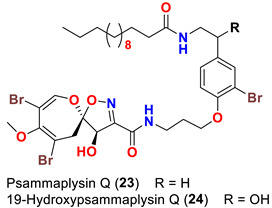	*Aplysinella strongyalata*	[50]
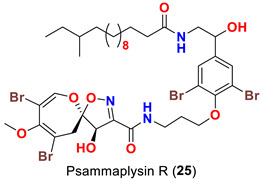	*Aplysinella strongyalata*	[50]
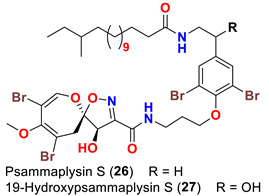	*Aplysinella strongyalata*	[50]
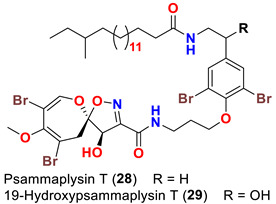	*Aplysinella strongyalata*	[50]
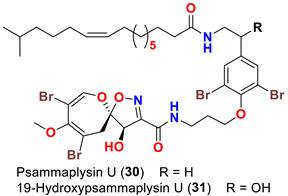	*Aplysinella strongyalata*	[50]
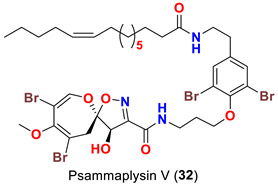	*Aplysinella strongyalata*	[50]
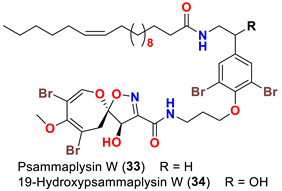	*Aplysinella strongyalata*	[50]
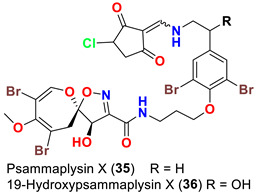	*Suberea* sp.	[51]
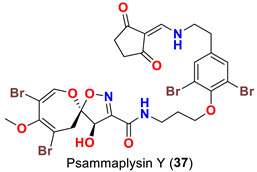	*Suberea* sp.	[51]
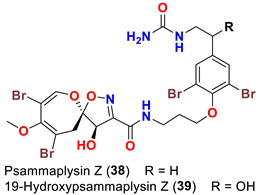	*Aplysinella* sp.	[41]
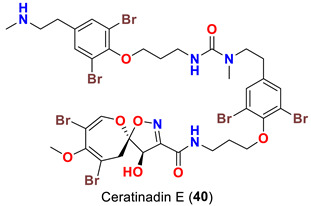	*Pseudoceratina* sp.	[52]
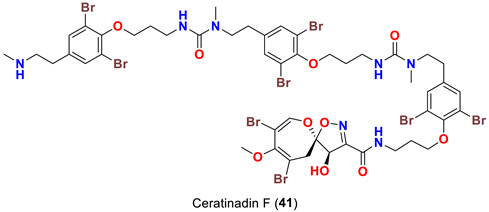	*Pseudoceratina* sp.	[52]
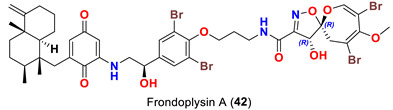	*Dysidea frondosa*	[53]
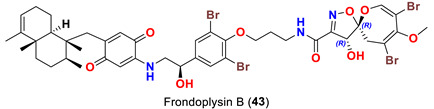	*Dysidea frondosa*	[53]
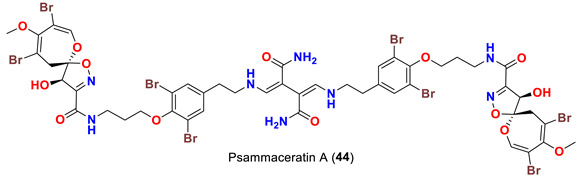	*Pseudoceratina arabica*	[54]

**Table 2:** Compounds with reported antimicrobial activities.

Compounds	Antimicrobial Activity	Reference
Psammaplysin A (**1**)	In vitro activity against gram positive bacteria and *E. coli* Antibacterial activity against *Flavobacterium marinotypicum* Inhibits mycothiol-S-conjugate amidase of *Mycobacterium tuberculosis* with IC_50_ of 20 µM	[39,45,56]
Psammaplysin B (**2**)	In vitro activity against Gram-positive bacteria and *E. coli* Inhibits mycothiol-S-conjugate amidase of *Mycobacterium tuberculosis* with IC_50_ of 26 µM	[39,56]
Psammaplysin F (**10**)	Inhibits the growth of *S. aureus* NCTC 6571, *S. aureus* 1H, *E. facecalis* NCTC-775, B. cereus NCTC-7464, MRSA MW2 and MRSA USA-30050 with MIC values of 42.8, 42.8, 42.8, 42.8, 40.0, and 80.0 µM	[57]
Psammaplysin (**12**)	Inhibits the growth of *S. aureus* NCTC 6571, *S. aureus* 1H, *E. facecalis* NCTC-775, B. cereus NCTC-7464, MRSA MW2 and MRSA USA-30050 with MIC values of 20.4, 45.0, 81.5, 81.5, 40.0, and 80.0 µM	[57]

**Table 3:** Compounds with reported cancer cells’ growth inhibition activities.

Compounds	Cytotoxicity	Reference
Psammaplysin A (**1**)	- Inhibits HCT-116 with an IC_50_ of 6 µg/mL - Inhibits HCT-15, PC-3, ACHN, MDA-MB-231, NUGC-3 and NCI-H23 with GI_50_ values 3.9, 6.9, 5.1, 4.3, 3.8, and 12.4 µM, respectively - Inhibits MDA-MB-231 and Hela cells with IC_50_ values of 2.9 and 8.5 µM respectively - Inhibits HCT116, MDA-MB-231, and Hela cells with IC_50_ values of 5.1, 3.90, and 8.50 respectively	[41,43,51,54]
Psammaplysin B (**2**)	- Inhibits HCT-116 with IC_50_ value of 6 µg/mL - Inhibited HCT-15, PC-3, ACHN, MDA-MB-231, NUGC-3, and NCI-H23 with GI_50_ values of 4.0, 2.7, 1.6, 0.53, 2.5, and 3.7 µM, respectively	[43,51]
Psammaplysin C (**3**)	Inhibits HCT-116 with IC_50_ of 3 µg/mL	[43]
Psammaplysin D (**4**)	- Weak growth inhibition of HCT-15, PC-3, ACHN, MDA-MB-231, NUGC-3, and NCI-H23 with GI_50_ values 24, 25, 27, 21, 26, and 27 µM, respectively	[51]
Psammaplysin E (**5**)	- Inhibited KB and LoVo cells at 5 µg/mL - Inhibited P388 cell with IC_50_ of 2.1 µg/mL - Inhibited HCT-15, PC-3, ACHN, MDA-MB-231, NUGC-3, and NCI-H23 cells with GI_50_ values 3.8, 1.4, 2.3, 0.51, 2.3, and 3.6 µM, respectively - Antimigratory activity against MDA-MB-231 and Hela cells with IC_50_ values of 0.29 and 2.1 µM, respectively	[41,44,45,51]
Psammaplysin F (**10**)	Inhibits HEK293 mammalian cell line with IC_50_ value of 11 μM	[47]
Psammaplysin X (**35**)	Inhibits HCT-15, PC-3, ACHN, MDA-MB-231, NUGC-3, and NCI-H23 cells with GI_50_ values of 3.3, 2.3, 3.3, 1.2, 3.5, and 6.4 µM, respectively	[51]
19-Hydroxypsammaplysin X (**36**)	Inhibits HCT-15, PC-3, ACHN, MDA-MB-231, NUGC-3, and NCI-H23 cells with GI_50_ values of 3.5, 2.1, 2.5, 0.8, 4.0, and 3.5 µM, respectively	[51]
Psammaplysin Z (**38**)	Inhibits MDA-MB-231 and Hela cell lines with IC_50_ values 19.4 and 22.2 µM, respectively	[41]
19-Hydroxypsammaplysin Z (**39**)	Inhibits MDA-MB-231 and Hela cell lines with IC_50_ values of 13.2 and 17.6 µM, respectively	[41]
Psammaceratin A (**44**)	Inhibits MDA-MB-231, Hela, and HCT116 cells with IC_50_ values of 5.25, 9.40, and 3.10 µM, respectively	[54]

**Table 4:** Compounds with reported antimalarial activities.

Compound	Activity	Reference
19-Hydroxypsammaplysin E (**6**)	Inhibits 3D7 chloroquine-sensitive strain of *P. falciparum* with IC_50_ of 6.4 μM	[50]
Psammaplysin F (**10**)	- Inhibits 3D7 and Dd2 strains of *P. falciparum* with IC_50_ values of 0.87 and 1.4 μM - Inhibits the drug-resistant (K1) and drug-sensitive (FCR3) strains of *P. falciparum* with IC_50_ values of 3.77 and 2.45 µg/mL and with selectivity indices of 3.4 and 5.2, respectively	[47,52]
Psammaplysin G (**11**)	Inhibits 98% of Dd2 cell strain of *P. falciparum* at 40 μM	[47]
Psammaplysin H (**12**)	Inhibits the 3D7 strain of *P. falciparum* with an IC_50_ value of 0.41 µM and selective towards the 3D7 strain of *P. falciparum* with a selectivity index (SI) of >97%	[48]
Ceratinadin E (**40**)	Inhibits K1 and FCR3 strains of *P. falciparum*, with IC_50_ values of 1.03 and 0.77 μg/mL, respectively and with selectivity indices (SI) of 15.5 and 20.8, respectively	[52]
Ceratinadin F (**41**)	Inhibits K1 strain of *P. falciparum* with an IC_50_ >12.5 μg/mL and selectivity index (SI) value of >4	[52]

**Table 5:** Compounds with reported antifouling activities.

Compound	Activity	Reference
Psammaplysin A (**1**)	Inhibits metamorphosis and settlement of *B. amphitrite* with an ED_50_ 0.27 µg/mL	[45]
Psammaplysin E (**5**)	Inhibits metamorphosis and settlement of *B. amphitrite* with an ED_50_ 4.8 µg/mL	[45]
Ceratinamide A (**7**)	Inhibits metamorphosis and settlement of *B. amphitrite* with an ED_50_ 0.10 µg/mL Induces metamorphosis on the ascidian *Halocynthia roretzi* with ED_100_ of 1.2 µg/mL	[45]
Ceratinamide B (**8**)	Inhibits metamorphosis and settlement of *B. amphitrite* with an ED_50_ 2.4 µg/mL	[45]

**Table 6:** Compounds with other reported activities.

Compound	Activity	Reference
Psammaplysin D (**4**)	Inhibits 51% of the Haitian RF strain of HIV-I at 0.1 µg/mL	[44]
Psammaplysin E (**5**)	Moderate immunosuppressive activity with a potency of 40, ICW = 8.323-01 for mixed lymphocyte reaction assay	[44]
Frondoplysin A (**42**)	- Inhibits protein tyrosine phosphatase 1B with an IC_50_ value of 0.39 μM compared to oleanolic acid as a positive control (IC_50_ 3.7 μM) and thiazolidinediones (IC_50_ 5.0 μM) similar to benzofuran and benzothiophene biphenyls (IC_50_ 0.36 μM) - Antioxidant activity in transgenic zebrafish without any cytotoxicity at 64 μM	[53]
Frondoplysin B (**43**)	Inhibits protein tyrosine phosphatase 1B with an IC_50_ value of 0.65 μM compared to oleanolic acid as a positive control (IC_50_ 3.7 μM)	[53]
Psammaplysin F (**10**) and its urea semisynthetic analogs **45**, **51**, **53** and **54**	Reduce the mitochondrial membrane potential (MMP)	[55]

## Data Availability

Not applicable.

## Supplementary Materials

The following supporting information can be downloaded at: https://www.mdpi.com/article/10.3390/md20110663/s1, Table S1. Reported compounds with 1,6-dioxa-2-azaspiro[4.6]undecane moiety.
