# Supplementary material for: Psammaplysins: Insights from Natural Sources, Structural Variations, and Pharmacological Properties
Source: Mar Drugs. 2022 Oct 25;20(11):663. doi: 10.3390/md20110663 (PMC9693029; doi:10.3390/md20110663)
Supplement: Supplementary file 1 [file marinedrugs-20-00663-s001.zip › marinedrugs-1949116-supplementary.pdf]

Table S1. Reported compounds with 1,6-dioxa-2-azaspiro[4.6]undecane moiety.

Compound	Sponge Name	Site of Collection	Optical Activity $[\alpha]_D$	Purification of the compound	Structural Assignment	Reference
Psammaplysin A (**1**)	*Psammaplysilla purpurea*	Gulf of Eilat, Red Sea		SiO_2 CC and Sephadex LH-20	UV, IR, ^1H,^{13}C-NMR, stepwise acetylation, and Elemental analysis	[39]
	Psammaplysilla purpurea	Near Au- gulpelu Reef, Palau, Western Caroline Islands	−65.2° (*c* 0.52, MeOH) at 22 °C	CC using Bio-Sil A 200-400 mesh) and Bio-Beads S-X8 and S-X12 (200-400 mesh) and Sephadex LH-20, SiO_2 TLC and KCl8F RP TLC plates	UV, ^1H,^{13}C-NMR, EIMS, 2D ^{13}C-^1H chemical shift correlation map, 2D ^{13}C-^{13}C connectivity plot and Single crystal X-ray diffraction	[40]
	Druinella (Psammaplysill) purpurea	Shallow reef waters off Makaluva Island of the Fiji Island Group in the South Pacific		Sephadex LH-20 CC and silica HPLC	IR, UV, ^1H, ^{13}C-NMR, and MS	[43]
	Aplysinella sp.	Pingelap Atoll, Micronesia		VLC, HPTLC, Preparative TLC, Sephadex LH-20 column, C18 and SiO_2 HPLC	UV, IR,^1H, ^{13}C-NMR and HRFABMS	[44]
	Pseudoceratina arabica	Sharm El-Sheikh, Egyptian Red Sea coast		SiO_2 CC, and Sephadex LH-20	UV, ^1H and ^{13}C NMR, COSY, HSQC, HMBC, and HRFABMS	[36]
	Aplysinella strongylata	Tulamben, Bali, Indonesia		SiO_2 CC and Semipreparative C18-HPLC	^1H, ^{13}C NMR, HMBC, HSQC, 1D TOCSY (+)-LRESIMS and (+)-HRESIMS	[50]
	Aplysinella sp.	Chuuk Atoll, Federated States of Micronesia.		SiO_2 VLC and SiO_2 HPLC	UV, ^1H- and ^{13}C-NMR ^1H-^1H COSY, HMQC, and HMBC	[46]
	Suberea sp.	Black Coral Kingdom, Guam	−48° (c 0.05, MeOH) at 25 °C	C18 VLC, and C18 HPLC	^1H, ^{13}C NMR, 2DNM, HRESIMS, IR, and UV	[49]
	Suberea sp.	Federated States of Micronesia	−71° (*c* 0.5, MeOH) at 25 °C	C18 CC, and C18 MPLC		[51]
	Aplysinella sp.	Off Jizan, Red Sea, Saudi Arabia		SiO_2, VLC, Sephadex LH-20, Sep-Pak C18, and C18 HPLC	Comparison of 1D, 2D NMR, and MS Spectra with the literatures	[41]
	Pseudoceratina purpurea	Hachijo-jima Island, Tokyo		SiO_2 CC, Gel permeation on Toyopearl HW-40, and C18 HPLC.	UV, IR, ^1H, ^{13}C-NMR, HMBC, and HRFABMS	[45]

	Pseudoceratina arabica	Anas Reef, Obhur, Red Sea, Saudi Arabia		SiO_2 VLC, Sephadex LH-20, and ODS HPLC	Comparison of 1D, 2D NMR, and MS Spectra with the literatures	[54]
Psammaplysin B (**2**)	*Psammaplysilla purpurea*	Gulf of Eilat, Red Sea		Separated from psammaplysin A by N-acetylation and CC	UV, IR, ^{1}H and ^{13}C-NMR	[39]
	Psammaplysilla purpurea	Near Au- gulpelu Reef, Palau, Western Caroline Islands Palau	−60.2° (*c* 0.632, MeOH) at 25 °C	CC using Bio-Sil A 200-400 mesh) and Bio-Beads S-X8 and S-X12 (200-400 mesh) and Sephadex LH-20, precoated SiO_2, TLC and KCl8F C18 plates	IR, UV, ^{1}H, ^{13}C-NMR, and EIMS	[40]
	Druinella (Psammaplysill) purpurea	Shallow reef waters off Makaluva Island of the Fiji Island Group in the South Pacific		Sephadex LH-20 CC and SiO_2 HPLC	IR, UV, ^{1}H, ^{13}C-NMR, and MS	[43]
	Aplysinella sp.	Pingelap Atoll, Micronesia		VLC, HPTLC, Preparative TLC , Sephadex LH-20 column , C18 and SiO_2 HPLC	UV, IR, ^{1}H, ^{13}C-NMR and HRFABMS	[44]
	Aplysinella strongylata	Tulamben, Bali, Indonesia		SiO_2 CC and Semipreparative C18-HPLC	^{1}H, ^{13}C NMR, HMBC, HSQC, 1D TOCSY, (+)-LRESIMS and (+)-HRESIMS	[50]
	Aplysinella sp.	Chuuk Atoll, Federated States of Micronesia		Flash SiO_2 CC and Diol HPLC	UV, ^{1}H- and ^{13}C-NMR, ^{1}H-^{1}H COSY, HMQC, and HMBC	[46]
	Suberea sp.	Black Coral Kingdom, Guam	−61.5 (*c* 0.04, MeOH) at 25 °C	C18 VLC and C18 HPLC	^{1}H, ^{13}C NMR, 2D NMR, HRESIMS, IR, and UV	[49]
	Suberea sp.	Federated States of Micronesia	−75° (*c* 0.5, MeOH) at 25 °C	C18 CC, RP MPLC, and C18 HPLC		[51]
Psammaplysin C (**3**)	*Druinella (Psammaplysilla) purpurea*	Shallow reef waters off Makaluva Island of the Fiji Island Group in the South Pacific	−57.1° (*c* 0.014, MeOH) at 23 °C	Sephadex LH-20 CC, and SiO_2 HPLC	IR, UV, ^{1}H, ^{13}C-NMR, and MS	[43]
	Aplysinella sp.	Chuuk Atoll, Federated States of Micronesia		Flash SiO_2 CC and Diol HPLC	UV, ^{1}H- and ^{13}C-NMR, ^{1}H-^{1}H COSY, HMQC, and HMBC	[46]
Psammaplysin D (**4**)	*Aplysinella* sp.	Pingelap Atoll, Micronesia	−80.3° (*c* 0.3, acetone) at 18 °C	VLC, HPTLC, Preparative TLC, Sephadex LH-20 column, C18 and SiO_2 HPLC	UV, IR, ^{1}H, ^{13}C-NMR and HRFABMS	[44]
	Aplysinella strongylata	Tulamben, Bali, Indonesia		SiO_2 CC and Semipreparative C18 HPLC	^{1}H, ^{13}C NMR, HMBC, HSQC, 1D TOCSY, (+)-	[50]

					LRESIMS, and (+)-HRESIMS	
Psammaplysin E (**5**)	*Aplysinella* sp.	Pingelap Atoll, Micronesia	−71.4° (acetone, *c* 2.8) at 18 °C	VLC, HPTLC, Preparative TLC, Sephadex LH-20 column, C18 and SiO_2 HPLC	UV, IR, ^1H, ^{13}C-NMR and HRFABMS	[44]
	Aplysinella strongylata	Tulamben, Bali, Indonesia		SiO_2 CC and Semipreparative C18 HPLC	^1H, ^{13}C NMR, HMBC, HSQC, 1D TOCSY, (+)-LRESIMS and (+)-HRESIMS	[50]
	Aplysinella sp.	Chuuk Atoll, Federated States of Micronesia.		SiO_2 VLC and SiO_2 HPLC	UV, ^1H- and ^{13}C-NMR, ^1H-^1H COSY, HMQC, and HMBC	[46]
	Aplysinella sp.	Off Jizan, Saudi Arabia, Red Sea		Successive fractionation on SiO_2, Sephadex LH-20, and Sep-Pak C18 cartridge columns, and C18 HPLC	Comparison of NMR, and MS Spectra with literature	[41]
	Pseudoceratina purpurea	Hachijo-jima Island, Tokyo		SiO_2 CC , Gel permeation on Toyopearl HW-40, and C18 HPLC.	UV, IR, ^1H, ^{13}C-NMR, HMBC, and HRFABMS	[45]
19-Hydroxypsammaplysin E (**6**)	*Aplysinella strongylata*	Tulamben, Bali, Indonesia	−79.6° (*c* 0.21, $CHCl_3$)	SiO_2 VLC, C18 HPLC and HPLC chiral column	^1H, ^{13}C NMR, HMBC, HSQC, 1D TOCSY, (+)-LRESIMS and (+)-HRESIMS	[50]
Ceratinamide A (**7**)	*Pseudoceratina purpurea*	Hachijo-jima Island, Tokyo	−89.7° (*c* 0.146, MeOH) at 24 °C	SiO_2 CC, Gel permeation on Toyopearl HW-40, and C18 HPLC	UV, IR, ^1H, ^{13}C-NMR, HMBC, and HRFABMS	[45]
Ceratinamide B (**8**)	*Pseudoceratina purpurea*	Hachijo-jima Island, Tokyo	−53.5° (*c* 0.263, acetone) at 24 °C	SiO_2 CC, Gel permeation on Toyopearl HW-40, and C18 HPLC	UV, IR, ^1H, ^{13}C-NMR, HMBC, and HRFABMS	[45]
19-Hydroxyceratinamide A (**9**)	*Suberea* sp.	Federated States of Micronesia	−62° (c 0.5, acetone) at 25 °C	C18 CC, C18 MPLC, and Cyano HPLC	UV, ^1H, ^{13}C NMR, HMBC, HSQC, (+)-LRESIMS and (+)-HRESIMS	[51]
Psammaplysin F (**10**)	*Aplysinella* sp.	Chuuk Atoll, Federated States of Micronesia	−62.3° (c 1.2, MeOH-CH_2Cl_2)	SiO_2 VLC, and Diol or SiO_2 HPLC	UV, 1H- and ^{13}C-NMR, ^1H-^1H COSY, HMQC, and HMBC	[46]
	Hyattella sp.	Queensland Australia	−62.3° (c 1.2, MeOH) at 25 °C	C18 CC, and C18 HPLC	UV, IR, ^1H, ^{13}C NMR, HSQC, HMBC, COSY, LRESIMS and HRESIMS	[47]
	Pseudoceratina sp.	Australia		Fractionation and C18 HPLC	Comparison with literature	[48]
	Pseudoceratina sp.	Okinawa, Japan		SiO_2 CC, C18 CC and C18 HPLC	UV, IR, ESIMS, HRESIMS, HSQC, ^1H,^{13}C NMR, ^1H-^1H COSY, and ECD	[52]
Psammaplysin G (**11**)	*Hyattela* sp.	Queensland Australia	−66° (c $CHCl_3$, *c* 0.1) at 25 °C	C18 CC, C18 HPLC, and Preparative TLC	UV, IR, ^1H, ^{13}C NMR, HSQC, HMBC, COSY,	[47]

					LRESIMS and HRESIMS	
	Pseudoceratina sp.	Australia		Fractionation and C18 HPLC	Spectroscopic data comparison with literature values	[48]
Psammaplysin H (**12**)	*Pseudoceratina* sp.	Australia	−63.8° (*c* 0.1 $CHCl_3$) at 25 °C	Fractionation and C18 HPLC	LRESIMS, ^1H, ^{13}C NMR, HSQC, HMBC, ^1H-^1H COSY and ROSY	[48]
Psammaplysin I (**13**)	*Suberea* sp.	Black Coral Kingdom, Guam	−90° (c 0.35, MeOH) at 25 °C	C18 VLC, and C18 HPLC	^1H, ^{13}C NMR, 2D NMR, HRESIMS, IR, and UV	[49]
Psammaplysin J (**14**)	*Suberea* sp.	Black Coral Kingdom, Guam		C18 VLC, and C18 HPLC	^1H, ^{13}C NMR, 2D NMR, HRESIMS, IR, and UV	[49]
Psammaplysin K (**15**)	*Aplysinella strongylata*	Tulamben, Bali, Indonesia	−16° (*c* 0.09, $CHCl_3$) at 22 °C	SiO_2 VLC, Semipreparative C18 HPLC, analytical HPLC, chiral column	^1H, ^{13}C NMR, HSQC, HMBC, (+)-LRESIMS and (+)-HRESIMS	[50]
Psammaplysin K dimethoxy acetal (**16**)	*Aplysinella strongylata*	Tulamben, Bali, Indonesia	−15° (*c* 0.03, $CHCl_3$) at 24 °C	SiO_2 VLC, Semipreparative C18 HPLC, analytical HPLC, chiral column	^1H, ^{13}C NMR, HSQC, HMBC, (+)-LRESIMS, and (+)-HRESIMS	[50]
Psammaplysin L (**17**)	*Aplysinella strongylata*	Tulamben, Bali, Indonesia	−65.6° (*c* 0.19, acetone) at 22 °C	SiO_2 VLC, Semipreparative C18 HPLC, analytical HPLC, chiral column	^1H, ^{13}C NMR, HSQC, HMBC, 1D TOCSY, (+)-LRESIMS, and (+)-HRESIMS	[50]
Psammaplysin M (**18**)	*Aplysinella strongylata*	Tulamben, Bali, Indonesia	−33 (*c* 0.05, acetone) at 22 °C	SiO_2 VLC, Semipreparative C18 HPLC, analytical HPLC, chiral column	^1H, ^{13}C NMR, HSQC, HMBC, 1D TOCSY, (+)-LRESIMS and (+)-HRESIMS	[50]
Psammaplysin N (**19**)	*Aplysinella strongylata*	Tulamben, Bali, Indonesia	−43° (*c* 0.01, $CHCl_3$) at 22 °C	SiO_2 VLC, Semipreparative C18 HPLC, analytical HPLC, chiral column	^1H, ^{13}C NMR, (+)-LRESIMS, (+)-HRESIMS and GC-MS	[50]
Psammaplysin O (**20**)	*Aplysinella strongylata*	Tulamben, Bali, Indonesia	−74° (*c* 0.08, $CHCl_3$) at 24 °C	SiO_2 VLC, Semipreparative C18 HPLC, analytical HPLC, chiral column	^1H, ^{13}C NMR, (+)-LRESIMS, (+)-HRESIMS and GC-MS	[50]
Psammaplysin P (**21**)	*Aplysinella strongylata*	Tulamben, Bali, Indonesia	−11° (*c* 0.09, $CHCl_3$) at 24 °C	SiO_2 VLC, Semipreparative C18 HPLC, analytical HPLC, chiral column	^1H, ^{13}C NMR, (+)-LRESIMS and (+)-HRESIMS	[50]
19-Hydroxypsammaplysin P (**22**)	*Aplysinella strongylata*	Tulamben, Bali, Indonesia	−74° (*c* 0.08, $CHCl_3$) at 24 °C	SiO_2 VLC, Semipreparative C18 HPLC, analytical HPLC, chiral column	^1H, ^{13}C NMR, (+)-LRESIMS and (+)-HRESIMS	[50]
Psammaplysin Q (**23**)	*Aplysinella strongylata*	Tulamben, Bali, Indonesia	−55° (*c* 0.01, $CHCl_3$) at 24 °C	SiO_2 VLC, Semipreparative C18 HPLC, analytical HPLC, chiral column	^1H, ^{13}C NMR, (+)-LRESIMS, (+)-HRESIMS and GC-MS	[50]
19-Hydroxypsammaplysin Q (**24**)	*Aplysinella strongylata*	Tulamben, Bali, Indonesia	−92 (*c* 0.03, $CHCl_3$) at 24 °C	SiO_2 VLC, Semipreparative C18 HPLC,	^1H, ^{13}C NMR, (+)-LRESIMS and (+)-HRESIMS	[50]

				analytical HPLC, chiral column		
Psammaplysin R (**25**)	*Aplysinella strongylata*	Tulamben, Bali, Indonesia	−88° (*c* 0.09, $CHCl_3$) at 22 °C	SiO_2 VLC, Semipreparative C18 HPLC, analytical HPLC, chiral column	^1H, ^{13}C NMR, (+)-LRESIMS and (+)-HRESIMS	[50]
Psammaplysin S (**26**)	*Aplysinella strongylata*	Tulamben, Bali, Indonesia	−98° (*c* 0.05, $CHCl_3$) at 22 °C	SiO_2 VLC, Semipreparative C18 HPLC, analytical HPLC, chiral column	^1H, ^{13}C NMR, (+)-LRESIMS and (+)-HRESIMS	[50]
19-Hydroxypsammaplysin S (**27**)	*Aplysinella strongylata*	Tulamben, Bali, Indonesia	−117° (*c* 0.18, $CHCl_3$) at 22 °C	SiO_2 VLC, Semipreparative C18 HPLC, analytical HPLC, chiral column	^1H, ^{13}C NMR, (+)-LRESIMS and (+)-HRESIMS	[50]
Psammaplysin T (**28**)	*Aplysinella strongylata*	Tulamben, Bali, Indonesia	−90° (*c* 0.04, $CHCl_3$) at 22 °C	SiO_2 VLC, Semipreparative C18 HPLC, analytical HPLC, chiral column	^1H, ^{13}C NMR, (+)-LRESIMS and (+)-HRESIMS	[50]
19-Hydroxypsammaplysin T (**29**)	*Aplysinella strongylata*	Tulamben, Bali, Indonesia	−133° (*c* 0.13, $CHCl_3$) at 22 °C	SiO_2 VLC, Semipreparative C18 HPLC, analytical HPLC, chiral column	^1H, ^{13}C NMR, (+)-LRESIMS and (+)-HRESIMS	[50]
Psammaplysin U (**30**)	*Aplysinella strongylata*	Tulamben, Bali, Indonesia	−92° (*c* 0.17, $CHCl_3$) at 22 °C	SiO_2 VLC, Semipreparative C18 HPLC, analytical HPLC, chiral column	^1H, ^{13}C NMR, (+)-LRESIMS and (+)-HRESIMS	[50]
19-Hydroxypsammaplysin U (**31**)	*Aplysinella strongylata*	Tulamben, Bali, Indonesia	−69° (*c* 0.19, $CHCl_3$) at 22 °C	SiO_2 VLC, Semipreparative C18 HPLC, analytical HPLC, chiral column	^1H, ^{13}C NMR, (+)-LRESIMS and (+)-HRESIMS	[50]
Psammaplysin V (**32**)	*Aplysinella strongylata*	Tulamben, Bali, Indonesia	−22° (*c* 0.03, $CHCl_3$) at 22 °C	SiO_2 VLC, Semipreparative C18 HPLC, analytical HPLC, chiral column	^1H, ^{13}C NMR, (+)-LRESIMS and (+)-HRESIMS	[50]
Psammaplysin W (**33**)	*Aplysinella strongylata*	Tulamben, Bali, Indonesia	−43° (*c* 0.04, $CHCl_3$) at 22 °C	SiO_2 VLC, Semipreparative C18 HPLC, analytical HPLC, chiral column	^1H, ^{13}C NMR, (+)-LRESIMS and (+)-HRESIMS	[50]
19-Hydroxypsammaplysin W (**34**)	*Aplysinella strongylata*	Tulamben, Bali, Indonesia	−84° (*c* 0.04, $CHCl_3$) at 22 °C	SiO_2 VLC, Semipreparative C18 HPLC, analytical HPLC, chiral column	^1H, ^{13}C NMR, (+)-LRESIMS and (+)-HRESIMS	[50]
Psammaplysin X (**35**)	*Suberea* sp.	Federated States of Micronesia	−64° (*c* 0.5, acetone) at 25 °C	C18 CC, C18 MPLC, and Cyano HPLC	UV, ^1H, ^{13}C NMR, HMBC, HSQC, (+)-LRESIMS and (+)-HRESIMS	[51]
19-Hydroxypsammaplysin X (**36**)	*Suberea* sp.	Federated States of Micronesia	−82° (*c* 0.5, acetone) at 25 °C	C18 CC, C18 MPLC, and Cyano HPLC	UV, ^1H, ^{13}C NMR, HMBC, HSQC, (+)-LRESIMS and (+)-HRESIMS	[51]
Psammaplysin Y (**37**)	*Suberea* sp.	Federated States of Micronesia	−77° (*c* 0.5, acetone) at 25 °C	C18 CC, C18 MPLC, and Cyano HPLC	UV, ^1H, ^{13}C NMR, HMBC, HSQC, (+)-LRESIMS and (+)-HRESIMS	[51]

Psammaplysin Z (**38**)	*Aplysinella* sp.	Off Jizan, Saudi Arabia, Red Sea	−54° (*c* 0.1, MeOH)	SiO_2 VLC, Sephadex LH-20, Sep-Pak C18, and C18 HPLC	HRESIMS, ^{1}H, ^{13}C-NMR, DEPT, HSQC, HMBC, and ^{1}H-^{1}H COSY	[41]
19-Hydroxypsammaplysin Z (**39**)	*Aplysinella* sp.	Off Jizan, Saudi Arabia, Red Sea	−61° (*c* 0.1, MeOH)	SiO_2 VLC, Sephadex LH-20, Sep-Pak C18, and C18 HPLC	HRESIMS, ^{1}H, ^{13}C-NMR, DEPT, HSQC, HMBC, and ^{1}H-^{1}H COSY	[41]
Frondoplysin A (**40**)	*Dysidea frondosa*	Yongxing Island in the South China Sea	−30.4° (c 0.23, MeCN) at 25 °C	SiO_2 VLC, C18 MPLC CC column and C18 HPLC	ESIMS, HRMS, ^{1}H, ^{13}C NMR, COSY, HSQC, HMBC, NOESY, CD, and X-ray crystallography	[53]
Frondoplysin B (**41**)	*Dysidea frondosa*	Yongxing Island in the South China Sea	−13.3° (c 0.15, MeCN) at 20 °C	SiO_2 VLC, C18 MPLC CC column and C18 HPLC	ESIMS, HRMS, ^{1}H, ^{13}C NMR, COSY, HSQC, HMBC, NOESY, and CD	[53]
Ceratinadin E (**42**)	*Pseudoceratina* sp.	Okinawa, Japan	−45.1° (*c* 0.48, MeOH) at 24 °C	SiO_2 CC, C18 CC, and C18 HPLC	UV, IR, ESIMS, HRESIMS, HSQC, ^{1}H,^{13}C NMR, ^{1}H-^{1}H COSY, and ECD	[52]
Ceratinadin F (**43**)	*Pseudoceratina* sp.	Okinawa, Japanes	−25° (*c* 0.18, MeOH) at 25 °C	SiO_2 CC, C18 CC, and C18 HPLC	UV, IR, ESIMS, HRESIMS, HSQC, ^{1}H,^{13}C NMR, ^{1}H-^{1}H COSY, and ECD	[52]
Psammaceratin A (**44**)	*Pseudoceratina arabica*	Anas Reef, Obhur, Saudi Arabia	−59° (*c* 0.1, MeOH) at 25 °C	SiO_2 VLC, Sephadex LH-20, and C18HPLC	HRESIMS, ^{1}H, ^{13}C NMR, DEPT, COSY, HSQC, HMBC, and NOESY	[54]
